# Microvesicles Are Associated with Early Veno Venous ECMO Circuit Change during Severe ARDS: A Prospective Observational Pilot Study

**DOI:** 10.3390/jcm12237281

**Published:** 2023-11-24

**Authors:** Christophe Guervilly, Giovanni Bousquet, Laurent Arnaud, Ines Gragueb-Chatti, Florence Daviet, Mélanie Adda, Jean-Marie Forel, Françoise Dignat-George, Laurent Papazian, Antoine Roch, Romaric Lacroix, Sami Hraiech

**Affiliations:** 1 Assistance Publique-Hôpitaux de Marseille, Hôpital Nord, Médecine Intensive Réanimation, 13015 Marseille, France; giovanni.bousquet@ap-hm.fr (G.B.); ines.gragueb-chatti@ap-hm.fr (I.G.-C.); florence.daviet@ap-hm.fr (F.D.); melanie.adda@ap-hm.fr (M.A.); jean-marie.forel@ap-hm.fr (J.-M.F.); antoine.roch@ap-hm.fr (A.R.); sami.hraiech@ap-hm.fr (S.H.); 2 Faculté de Médecine, Centre d’Etudes et de Recherches sur les Services de Santé et Qualité de vie EA 3279, Aix-Marseille Université, 13005 Marseille, France; 3 Laboratoire d’Hématologie et de Biologie Vasculaire, Assistance Publique-Hôpitaux de Marseille, 13005 Marseille, France; laurent.arnaud@ap-hm.fr (L.A.); francoise.dignat-george@univ-amu.fr (F.D.-G.); romaric.lacroix@univ-amu.fr (R.L.); 4 INSERM 1263, Institut National de Recherche pour l’Agriculture, l’Alimentation et l’Environnement (INRAE), Centre de Recherche en CardioVasculaire et Nutrition (C2VN), Aix-Marseille Université, 13013 Marseille, France; 5 Centre Hospitalier de Bastia, Service de Réanimation, 604 Chemin de Falconaja, 20600 Bastia, France; laurent.papazian@ch-bastia.fr

**Keywords:** veno venous ECMO, ECMO circuit change, microvesicles, thrombosis, hemolysis

## Abstract

Background: Veno venous Extra Corporeal Membrane Oxygenation (vvECMO) is associated with frequent hematological ECMO-related complications needing ECMO circuit change. Microvesicles (MVs) interplay during the thrombosis-fibrinolysis process. The main objective of the study was to identify subpopulations of MVs associated with indications of early vvECMO circuit change. Methods: This is a prospective observational monocenter cohort study. Blood gas was sampled on the ECMO circuit after the membrane oxygenator to measure the PO_2 post oxy_ at inclusion, day 3, day 7 and the day of ECMO circuit removal. Blood samples for MV analysis were collected at inclusion, day 3, day 7 and the day of ECMO circuit removal. MV subpopulations were identified by flow cytometry. Results: Nineteen patients were investigated. Seven patients (37%) needed an ECMO circuit change for hemolysis (n = 4), a pump thrombosis with fibrinolysis (n = 1), persistent thrombocytopenia with bleeding (n = 1) and a decrease of O_2_ transfer (n = 1). Levels of leukocyte and endothelial MVs were significantly higher at inclusion for patients who thereafter had an ECMO circuit change (*p* = 0.01 and *p* = 0.001). The areas under the received operating characteristics curves for LeuMVs and EndoMVs sampled the day of cannulation and the need for ECMO circuit change were 0.84 and 0.92, respectively. PO_2 post oxy_ did not significantly change except for in one patient during the ECMO run. Conclusions: Our pilot study supports the potential interest of subpopulations of microvesicles early associated with hematological ECMO-related complications. Our results warrant further studies.

## 1. Background

Veno venous extracorporeal membrane oxygenation (vvECMO) is a valuable therapeutic option for severe acute respiratory distress syndrome (ARDS) unresponsive to protective lung ventilation, associated with prone positioning [1]. To counterbalance its benefits, ECMO also carries a burden of severe complications including bleeding and clotting which can occur simultaneously. During ECMO, severe bleeding events are associated independently with a worse prognosis, and a closed monitoring of coagulation parameters is suggested [2,3].

Hemolysis due to shear stress in the ECMO circuit is also a well-recognized adverse event of prolonged ECMO with a negative impact on prognosis [4]. Hemolysis may be a consequence of thrombosis occurring in centrifugal pumps and may also be a favoring factor for thrombosis [5]. In these cases, ECMO circuit changes are usually needed to break the vicious circle of hemostatic complications. To date, there is no definite biomarker indicating ECMO circuit change. 

Microvesicles (MVs) are a well-known major player in the process of thrombosis in inflammatory and infectious diseases, notably in the critically ill context [6,7]. In animal models of ARDS, the injection of human MVs released by endothelial cells have been implicated in the development of endothelial injury and lung injury [8]. In humans, high levels of MVs of leukocyte origin have been associated with better outcomes in ARDS patients [9]. During the coronavirus disease of 2019, MVs with tissue factor activity were strongly associated with thromboembolic event occurrence [10]. 

The generation of MVs during extracorporeal circulation has been previously described in experimental and clinical settings [11,12]. During veno-arterial ECMO, levels of MVs increased and subpopulations were modified. A higher ECMO blood flow could be associated with cellular activation in the extracorporeal circuit and the generation of MVs [13]. 

To date, there is no available data on MV phenotypes during vvECMO. The main objective was to identify early on the subpopulations of MVs associated with indications of vvECMO circuit change (e.g., thrombosis, hemolysis or a decrease in O_2_ transfer through the membrane). The second objective was to identify the subpopulations of MVs associated with ECMO survival. 

## 2. Methods

### 2.1. Study Design

This was a prospective observational pilot study conducted in a university ECMO center in Marseille, France. According to the French law, the study was approved by the ethics committee (Comité de Protection des Personnes Sud Méditerranée V) and written informed consent was obtained from the patients’ next of kin. The study was registered in the ClinicalTrial.gov database with the identifier NCT02879344. All adult patients admitted with severe ARDS [14] fulfilled vvECMO criteria [1] or, referred for vvECMO, were screened for enrollment in the study [15]. An attempt at prone positioning for at least 8–10 h was always considered before ECMO. To investigate the effect of ECMO duration on MVs levels, patients were included during a 6 h time frame after cannulation. In our center, the usual contra-indications for vvECMO are severe multi organ failure scored with a sequential failure assessment score (SOFA) [16] > 17 for patients with less than 7 days of mechanical ventilation and >11 for patients with more than 7 days of mechanical ventilation and heparininduced thrombocytopenia. Non-inclusion criteria were age <18, pregnancy, and person deprived of liberty or subject to legal protection measures. 

### 2.2. ECMO Management 

We used mostly the right femoral–right jugular veins configuration using an ultrasound-guided and percutaneous Seldinger approach with large bore cannulas of 27–29 F for the inflow to achieve ECMO blood flow of 4–6 L per minute, centrifugal pumps (Bio-console 560; Medtronic Perfusion Systems, Minneapolis, MN, USA), prolonged heparin-coated tubing and Quadrox D oxygenators (Getinge, Maquet, Goteborg, Sweden) to achieve arterial blood O_2_ saturation of 90–95%, while oxygen fraction delivered by the oxygenator was set at 1. Revolutions per minute (RPM) of the centrifugal pump were adjusted to achieve ECMO flow of at least 60% of cardiac output or more (evaluated by trans-thoracic or trans-esophageal echocardiography). Sweep gas flow of oxygen was progressively increased to achieve a pH value > 7.3 and PaCO_2_ < 45 mmHg. A heater-cooler unit was systematically incorporated into the ECMO circuit to ensure patient normothermia.

### 2.3. Anti-Coagulation Management and Transfusions Thresholds 

Continuous heparin infusion maintained activated partial thromboplastin time (aPTT) between 40 and 55 s. In case of severe bleeding and/or spontaneous prolonged aPTT, heparin infusion was sustained. Triggering limits for transfusion were 8 g/dL for hemoglobin and 50 Giga/L for platelets. Antithrombin (AT) substitution was indicated in case of deficiency (<60% of AT activity). Fibrinogen substitution was indicated to target a fibrinogen level ≥ 2 g/L.

### 2.4. Mechanical Ventilation Management 

Ventilator was set in volume-controlled mode with tidal volume (VT) of 2–4 mL/kg of predicted body weight, and a respiratory rate was set between 10 and 15 cycles per minute, with a PEEP ≥ 10 cmH_2_O to achieve plateau pressure ≤ 25 cmH_2_O and driving pressure ≤ 15 cmH_2_O. For VT ≤ 100 mL, we used the pediatric mode with dedicated circuit. We systematically used humidifier heater. Tracheal suctioning was performed with closed systems to prevent alveolar derecruitment. Minimal FiO_2_ was set on the ventilator to avoid alveolar oxidative stress. After improvement of lung function and respiratory system compliance, during the weaning process of vvECMO, patients were switched to partial assist modes such as airway pressure release ventilation (APRV) or pressure support ventilation (PSV) until mechanical ventilation weaning. Patients were switched from volume-controlled mode to APRV just after interruption of neuromuscular blocker to promote early spontaneous breathing ventilation. Then, switch to PSV was encouraged after decreasing sedation and improvement of respiratory system compliance. In APRV main settings were high pressure 25 cm of H_2_O, low pressure as previous PEEP set, inspiratory time of 1 s, expiratory time of 2 s. In PSV, pressure support was set to ensure tidal volume between 4 and 8 mL/kg/PBW without exceeding 15 cm of H_2_O of dynamic driving pressure. 

### 2.5. Sedation and Neuromuscular Blockers Utilization

During the first 48 hours of vvECMO run, patients received continuously combination of midazolam and opioids to achieve a RASS score ≤ −4. Neuromuscular blockers (NMB) were systematically continuously administered during the first 48 h following cannulation with targeting zero twitch at train-of-four testing. Thereafter, a first attempt to interrupt NMB was performed. After ECMO weaning, sedation was decreased to target RASS score of 0, −1 in order to promote spontaneous respiratory cycles.

### 2.6. Indications for ECMO Circuit Change

Indications for ECMO circuit change within our institution are intravascular hemolysis, bleeding with severe coagulation abnormality related to ECMO (e.g., thrombocytopenia < 50 G/L, fibrinolysis with fibrinogen < 1 g/L), ECMO circuit thrombosis and decrease in O_2_ transfer though the ECMO membrane.

### 2.7. Blood Samples and Microparticles Cytometry Analysis

Blood gases corrected for temperature were sampled on the ECMO circuit after the membrane oxygenator to measure the PO_2 post oxy_ at inclusion, day 3, day 7 and the day of ECMO circuit remove. A value of PO_2 post oxy_ < 200 mmHg indicated oxygenator change. Blood samples for MVs analysis were collected in citrate from an indwelling arterial catheter at inclusion, day 3, day 7 and the day of ECMO circuit removal. They were immediately centrifuged at 1500× *g* for 15 min to isolate plasma, and at 13,000× *g* for 2 min to remove residual platelets. Free platelet plasma obtained were thereafter stored at −80 °C until analysis. MVs were enumerated by flow cytometry as previously described [17]. Fluorescent antibody reagents used were procured from Beckman Coulter (Villepinte, France): CD31-PE, clone 1F11; CD41-PE-cyanin 7 (CD41-PC7), clone P2; CD11b-APC, clone Bear1; and CD235a-allophycocyanin (APC)-A750, clone KC16, as well as their respective controls: IgG1, clone 679.1Mc7. These reagents were used at their optimal final concentrations. All the isotype controls were matched with their relevant antibody conjugates in terms of final concentrations and fluorescence backgrounds. Annexin V-FITC was from Beckman Coulter. Briefly, 30 µL of PFP was incubated with the appropriate amount of each antibody or its isotype control plus 10 µL of AnnV-FITC. Each stained sample was analyzed on a NAVIOS 3-lasers instrument (Beckman-Coulter, Miami, FL, USA), following a protocol standardized with Megamix-Plus FSC beads (BioCytex, Marseille, France). Platelet-(PlatMVs), erythrocyte-(ErythroMVs), leucocyte-(LeucoMVs) and endothelial-(EndMVs) derived MPs were defined as AnnV+/CD41+, AnnV+/CD235a+, AnnV+/CD11b+ or AnnV+/CD31+/CD41− events, respectively. The absolute MV counts (events per µL) were determined using ad hoc counting beads (CytoCount, Dako, Copenhagen, Denmark). 

### 2.8. Statistical Analysis

Due of the exploratory characteristic of the study, we did not perform a sample-size calculation and planned to include 20 patients (protocol summary in ClinicalTrial.gov NCT02879344). Continuous variables were presented as mean ± sd and compared using Student’s two-tailed t-test. Normality of the distribution for the variables was assessed using the Kolmogorov–Smirnov test. Non-normally distributed continuous variables were presented as median and interquartile range and compared using Wilcoxon’s rank sum test. The chi-square test or the Fisher exact test was used to compare categorical variables. Received operating characteristic (ROC) curves were constructed to evaluate the value of MVs at inclusion for prediction of ECMO circuit change and death. Areas under the ROC curves (AUC) were determined and cut off values exhibited the best sensitivity, specificity, positive and negative likelihood ratios (LR+ and LR−) were calculated. A two tailed *p* ≤ 0.05 was considered statistically significant. Statistics and figures were performed with SPSS 20.0 (SPSS Inc. Chicago, IL, USA).

## 3. Results

### 3.1. Patients

During an eighteen months period, 34 adult patients requiring vvECMO for severe ARDS were screened for inclusion in the study. Among them, 14 were not included, 7 refused to participate, 6 for organizational reasons and 1 for pregnancy. The first patient was enrolled on 14 January 2013, and the last on 24 June 2014. The last patient’s outcome was obtained on 30 July 2014. We obtained 20 informed consents but due to an early death, only 19 patients were able to have blood samples for MV dosages and these were analyzed. The main characteristics and outcomes are displayed in Table 1. Table 2 compares ECMO characteristics at inclusion between patients who died under ECMO and those who were weaned alive from ECMO. 

### 3.2. Membrane Oxygenator Efficiency during ECMO Run

**Figure 1:**
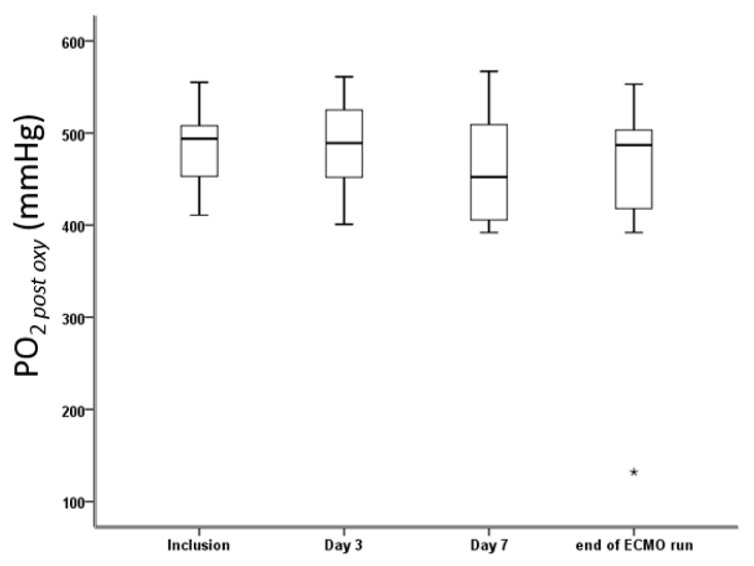
Evolution of PO_2 post oxy_ genator during ECMO runs. * means extreme value.

The mean post-oxygenator PO_2_ was 472 ± 65 mmHg during the study. Concerning membrane oxygenator efficiency, we did not observe a significant decrease in PO_2 post oxy_ during the ECMO run except for one patient with PO_2 post oxy_ at 132 mmHg after 21 days with the same membrane oxygenator, whereas PO_2 post oxy_ at inclusion was 506 mmHg (Figure 1).

### 3.3. ECMO Circuit Change and Subpopulations of MPs

**Figure 2:**
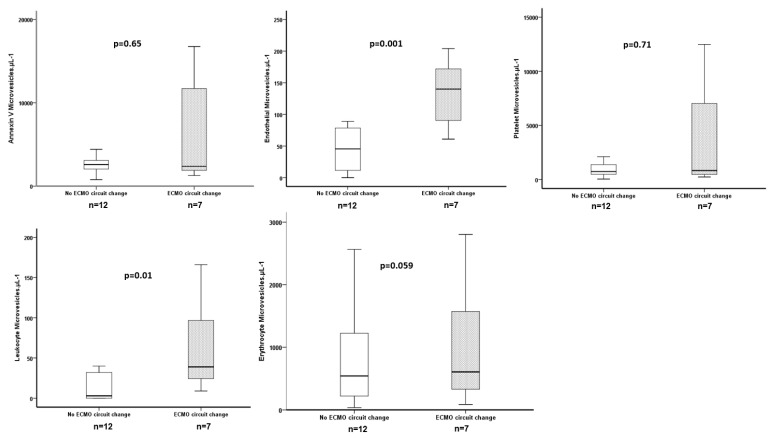
Levels of total MVs and MVs subpopulations at inclusion and need for an ECMO circuit change.

**Figure 3:**
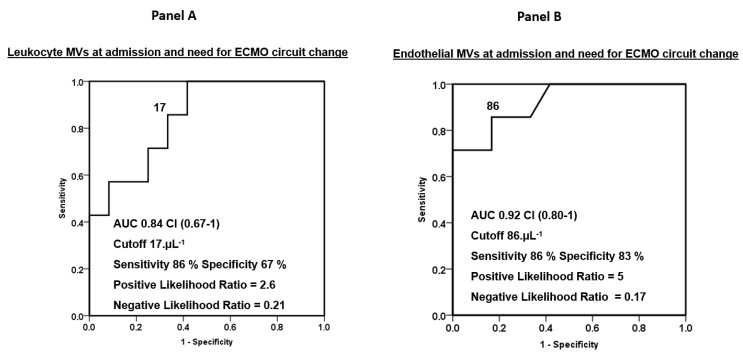
ROC curves for leukocyte (panel (**A**)) and endothelial (panel (**B**)) MVs measured at admission and need for an ECMO circuit change.

**Figure 4:**
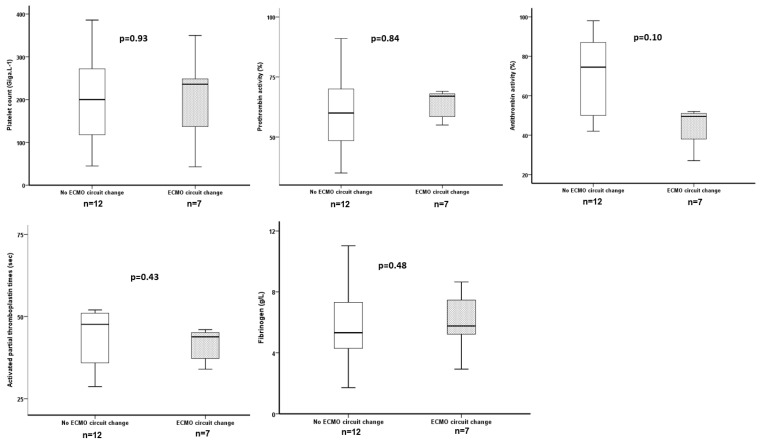
Comparisons of usual coagulation parameters measured at inclusion and need for an ECMO circuit change during the ECMO run.

Seven patients (37%) needed an ECMO circuit change for intravascular hemolysis (n = 4), pump thrombosis with fibrinolysis (n = 1), persistent thrombocytopenia with bleeding (n = 1) and a decrease in O_2_ transfer (n = 1). The levels of leukocyte and endothelial MVs were significantly higher at inclusion for patients who thereafter had an ECMO circuit change (respectively, *p* = 0.01 and *p* = 0.001) (Figure 2). The ROC curves for LeuMVs and EndoMVs and the need for ECMO circuit change are represented in Figure 3, with AUCs of 0.84 and 0.92 (panels A and B, respectively). Concerning usual coagulation parameters, we did not find a significant difference at inclusion between patients who thereafter had an ECMO circuit change (Figure 4).

### 3.4. Outcomes and Subpopulations of MVs

Twelve patients (63%) were weaned alive from ECMO. Among them, 11 (58%) were ICU-survivors. Eight patients died, seven during ECMO and one after decannulation. Their causes of death were refractory septic shock (4 cases), intractable pulmonary bleeding (2 cases), brain hemorrhage (1 case) and pulmonary fibrosis (1 case). 

**Figure 5:**
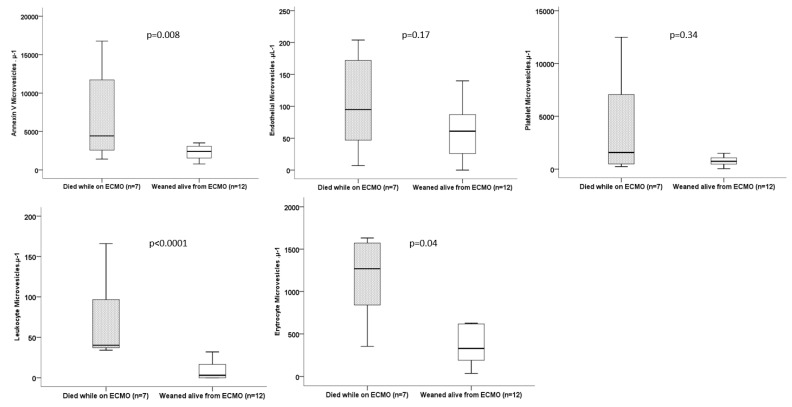
Comparison of MVs levels at inclusion and outcomes.

**Figure 6:**
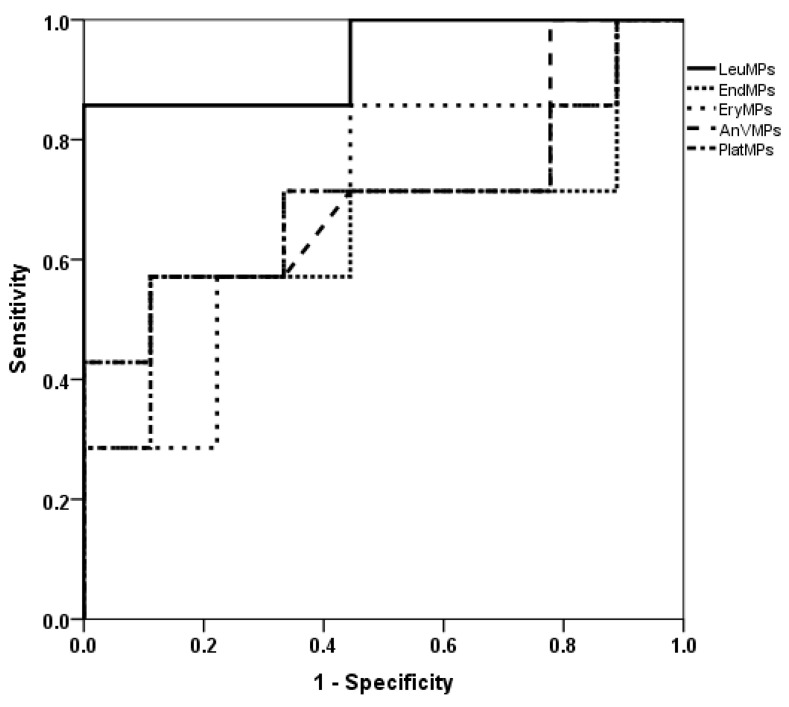
ROC curves for total MVs and subpopulations measured at admission and risk of death. LeuMVs AUC 0.94 CI (0.81–1), *p* = 0.001 vs. 0.5. cutoff 33 µL^−1^; sensitivity 87%, specificity 100%, LR+ = 87, LR− = 0.13.

LeuMVs and ErythroMVs were significantly higher at inclusion in patients who thereafter died while on ECMO (*p* < 0.001 and *p* = 0.04, respectively) (Figure 5). ROC curves for the risk of death for subpopulations of MVs showed the greatest AUCs for LeuMVs [0.94, CI (0.81–1)], with a cutoff value of 33 µL^−1^ exhibiting a sensibility of 87%, a specificity of 100%, a positive likelihood ratio of 87 and a negative likelihood ratio of 0.13 (Figure 6). Concerning the usual coagulation and hematological parameters, there was no difference at inclusion except for a lower prothrombin activity in patients who died thereafter on ECMO support (Table 3).

## 4. Discussion

The present study illustrates the interplay between circulating MVs from various cellular origins, extracorporeal membrane circuit complications and the prognosis of patients presenting with severe ARDS and supported by vvECMO. The main results are the following, 1/we were not able to demonstrate a decrease in O_2_ transfer in the ECMO membrane along time with the limit of relatively short ECMO runs with the same membrane (median 12 days), 2/more than one patient out of three had presented ECMO-related complications requiring ECMO circuit changes and 3/we found among MVs some subpopulations which were associated either with a need for circuit change, or with vvECMO weaning. Concerning prognosis, LeuMVs were found to be strongly associated with death during vvECMO.

During vvECMO, circuit changes were frequent, with range from 16% to 31% described [18,19], and could be associated with worse outcomes [20]. 

Hemocompatibility was one of the cornerstones of extracorporeal circuit tolerance, particularly in the field of long-term use such as for vvECMO. Despite treatment surfaces and careful systemic anticoagulation use, a significant percentage of patients developed thrombotic complications and/or hemorrhagic syndromes [21] with possible involvement of MV generations [12]. Therefore, a comprehensive physiopathology of the imbalance between pro and anticoagulation mechanisms is crucial in this setting to perform personalized coagulation activation monitoring. 

Progressive or acute clot formations in the circuit (pump head and/or membrane lung) were frequent events with potential negative impacts on ECMO duration and costs [19]. Therefore, the early detection of these fibrin-platelet thrombi seems essential in order to improve the efficiency of the technique and to reduce morbidity and costs. To date, there is no reliable marker predictive of the formation of these thrombi. Sophisticated tools such as multi-computed tomography for visualization and quantification of ex vivo clots [22,23] and the blood D-dimer assay were studied. Both of them present limiting factors. D dimers assay exhibits a low specificity, with risings in multiple concomitant pathologies, such as inflammation, cancers, pregnancy and trauma. Alternatively, the use of a multi-computed tomography detector requires the ex vivo fragmentation of the membranes to visualize the clots, not allowing screening for thrombus formation but for an a posteriori diagnosis.

Beside the sample size and single center design, our study presents some limitations.

First, MV identification requires expertise and specific technology not widely spread in every hematological lab. Second, we did not measure MVs after circuit change to assess their kinetic. Third, we did not investigate innovative biomarkers of thrombosis/fibrinolysis as tissue factor pathway inhibitors, plasminogen activator inhibitor-1 or soluble thrombomodulin. Finally, our results may need to be balanced by the relatively high rate of ECMO circuit changes observed in our cohort.

## 5. Conclusions

Our pilot study supports the potential interest of subpopulations of microvesicles associated with hematological ECMO-related complications. Our results warrant further studies including a functional assay of subpopulations of MVs and tissue factor activity measurement.

## Figures and Tables

**Table 1:** Characteristics of the population.

Variables	N = 19
Age (years)	3 ± 17
Gender (male), n (%)	14 (74)
Body mass index (kg·m^−2^)	27 ± 6
Simplified Acute Physiologic Score II at admission	46 ± 14
Sequential Organ Failure Assessment Score at inclusion	10 ± 3
ARDS main risk factor, n	
Bacterial pneumonia	10
Viral pneumonia	2
Aspiration pneumonia	2
Grade 3 primary graft dysfunction following lung transplantation	2
Alveolar hemorrhage	1
Pancreatitis	1
Peritonitis	1
ECMO duration (days)	12 ± 7
ECMO circuit change, n (%)	7 (37)
Weaning from ECMO, n (%)	12 (63)
Mechanical ventilation duration (days)	25 ± 17
ICU length of stay (days)	25 ± 17
ICU survival, n (%)	11 (58)
Blood products consumption during ECMO run	
Red packed blood cells	11 ± 9
Plasma concentrates	1.4 ± 2.8
Platelet concentrates	1.4 ± 1.8
Fibrinogen, g	0.4 ± 1.4
Recombinant antithrombin III, IU	950 ± 2275

**Table 2:** ECMO characteristics at inclusion.

	All Population N = 19	Died While on ECMO N = 7	Weaned Alive from ECMO N = 12	*p* Value
Configuration				1
Femoro-jugular, n (%)	18 (95)	7 (100)	11 (92)	
Femoro-femoral, n (%)	1 (5)	0 (0)	1 (8)	
ECMO flow, L·min^−1^	4.7 (4–4.9)	4.4 (3.9–4.9)	4.7 (4.4–5)	0.2
Revolutions per minute	2830 (2625–3080)	2740 (2680–3130)	2885 (2437–3067)	0.8
Sweep gas flow, L·min^−1^	6 (5–7)	6 (5–6.5)	6 (5–7)	0.8
PaO_2 post oxy_ (mmHg)	494 (453–510)	513 (488–538)	471 (450–499)	0.08

**Table 3:** Coagulation, hematological and biochemical data at inclusion.

	All Population N = 19	Died While on ECMO N = 7	Weaned Alive from ECMO N = 12	*p* Value
Leucocytes, Giga/L	18 (10–24)	20 (10–36)	16 (10–24)	0.5
Red blood cells, Tera/L	3 (2.8–3.6)	3 (2.9–3.3)	3.2 (2.6-3.6)	0.9
Hemoglobin, g/dL	9.1 (8.4–10.3)	8.7 (8.4–9)	9.7 (8.3–11.1)	0.2
Platelet, Giga/L	206 (111–275)	170 (45–333)	213 (114–258)	0.7
Prothrombin activity, %	62 (49–69)	55 (39–66)	68 (53–70)	0.04
Fibrinogen, g/L	5.5 (4.3–7.6)	5.2 (2.4–6.4)	6.4 (4.6–8.2)	0.07
aPTT, s	45 (36–50)	38 (34–52)	45 (40–50)	0.6
Antithrombin activity, %	51 (48–84)	50 (34–85)	52 (49–87)	0.4
lactate dehydrogenase (IU/L)	1348 (876–2550)	2550 (647–3818)	1309 (928–2222)	0.5
Lactate, mmol/L	2.4 (1.6–5.2)	2.4 (1.5–5.8)	2.2 (1.6–4.9)	0.7
Procalcitonin, µg/L	1.8 (0.8–5.4)	1.8 (1.1–19)	2.4 (0.4–5.2)	0.6

ECMO, extracorporeal membrane oxygenation; aPTT, activated partial thromboplastin time; IU, international unit.

## Data Availability

The datasets used and analyzed during the current study are available from the corresponding author on reasonable request.
